# Association between Prostate Cancer and Urinary Calculi: A Population-Based Study

**DOI:** 10.1371/journal.pone.0057743

**Published:** 2013-02-25

**Authors:** Shiu-Dong Chung, Shih-Ping Liu, Herng-Ching Lin

**Affiliations:** 1 Division of Urology, Department of Surgery, Far Eastern Memorial Hospital, New Taipei City, Taiwan; 2 School of Health Care Administration, Taipei Medical University, Taipei, Taiwan; 3 Department of Urology, National Taiwan University Hospital and College of Medicine, Taipei, Taiwan; University of Kentucky College of Medicine, United States Of America

## Abstract

**Background:**

Understanding the reasons underlying the emerging trend and the changing demographics of Asian prostate cancer (PC) has become an important field of study. This study set out to explore the possibility that urinary calculi (UC) and PC may share an association by conducting a case-control study on a population-based database in Taiwan.

**Methods:**

The cases of this study included 2,900 subjects ≥ 40 years-old who had received their first-time diagnosis of PC and 14,500 randomly selected controls without PC. Conditional logistic regressions were employed to explore the association between PC and having been previously diagnosed with UC.

**Results:**

We found that prior UC was found among 608 (21.0%) cases and 2,037 (14.1%) controls (*p*<0.001). Conditional logistic regression analysis revealed that compared to controls, the odds ratio (OR) of prior UC for cases was 1.63 (95% CI = 1.47–1.80). Furthermore, we found that cases were more likely to have been previously diagnosed with kidney calculus (OR = 1.71; 95% CI = 1.42–2.05), bladder calculus (OR = 2.06; 95% CI = 1.32–3.23), unspecified calculus (OR = 1.66; 95% CI = 1.37–2.00), and ≥2 locations of UC (OR = 1.73; 1.47–2.02) than controls. However, there was no significant relationship between PC and prior ureter calculus. We also found that of the patients with UC, there was no significant difference between PC and treatment method.

**Conclusions:**

This investigation detected an association between PC and prior UC. These results highlight a potential target population for PC screening.

## Introduction

Urinary Calculi (UC) is a common genitourinary disorder that has had an increasing incidence over the past 100 years and is currently estimated to occur during the lifetimes of 10–15% of the global population [1]–[4]. Therefore, on account of the relatively high and increasing incidence rate of UC, it is important to understand what conditions may be associated with the many survivors of this low-mortality condition.

Prostate is the third most common cancer in the world with an age-standardised rate of 104 per 100,000, and is the most frequently diagnosed male cancer in the United States [5]. Although prostate cancer (PC) is considerably less prevalent in Asian countries than in the west, with Chinese men living in the Far East developing clinically apparent prostate cancer at a rate nearly one-tenth that of Caucasian men living in Western countries [6]–[8], the incidence of PC in the East is on the rise [9], [10]. Understanding the reasons underlying this emerging trend and the changing demographics of Asian PC has become an important field of study, with several epidemiological studies involving multi-disciplinary and multi-centre collaboration currently investigating suspected environmental factors [10]. As dietary factors have been linked to both UC and PC, this study set out to explore the possibility that UC and PC may share an association by conducting a case-control study on a nationwide population-based database in Taiwan.

## Methods

### Database

This study used data retrieved from the Taiwan Longitudinal Health Insurance Database 2000 (LHID2000). Taiwan initiated the National Health Insurance (NHI) program in March 1995 to finance health care for all citizens of Taiwan. The LHID2000 includes the medical claims data for 1,000,000 beneficiaries, randomly sampled from the year 2000 registry of NHI beneficiaries (*n* =  23.72 million). The Taiwan National Health Research Institute has validated the representativeness of the LHID2000, and confirmed that it corresponds with the whole population of NHI beneficiaries on sex and age distribution. In addition, prior studies have demonstrated the high validity of the data from the NHI program [11], [12]. Many researchers have utilized the LHID2000 to perform and publish studies in internationally peer-reviewed journals [13], [14].

This study was exempted from full review by the Institutional Review Board (IRB) of the Taipei Medical University after consulting with the director of the Taipei Medical University IRB since the LHID2000 consists of de-identified secondary data released to the public for research purposes.

### Study Population

To select the cases used in this case-control study, we first identified 2,900 subjects ≥ 40 years-old who had received their first-time diagnosis of PC (ICD-9-CM code 185, malignant neoplasm of prostate) during ambulatory care visits between January 2002 and December 2009. We assigned the date of their first PC diagnosis as their index date. We also assured that no selected cases had ever received a diagnosis of PC prior to their index date. Furthermore, in order to increase the diagnostic validity of PC in this study, we only included PC subjects who had received at least two PC diagnoses during the period between 2002 and 2009 interspersed by a maximum of one month.

We further selected five controls for each case from the remaining beneficiaries in the LHID2000. The 14,500 controls were frequency-matched with cases with regard to age group (40–49, 50–59, 60–69, 70–79, and >79), urbanization level of each subject’s residence (5 levels, with 1 meaning the most urbanized and 5 the least), geographic region (Northern, Central, Eastern, and Southern Taiwan) and index year. We confirmed that none of the selected controls had a history of PC. For controls, we assigned their first use of medical services occurring in the index year as their index date. As a result, 17,400 subjects were included in this study.

### Exposure assessment

We identified the UC cases based on ICD-9-CM codes 592 (calculus of kidney and ureter), 592.0 (calculus of kidney), 592.1 (calculus of ureter), 592.9 (urinary calculus, unspecified), 594 (calculus of lower urinary tract), 594.0 (calculus in diverticulum of bladder), 594.1 (other calculus in bladder), 594.2 (calculus in urethra) 594.8 (other lower urinary tract calculus), and 594.9 (calculus of lower urinary tract, unspecified). In order to increase diagnostic validity, we only selected patients who had received two or more UC diagnoses prior to the index date, with at least one being made by a urologist or nephrologist.

### Statistical Analysis

The SAS system (SAS System for Windows, Version 8.2, SAS Institute Inc, Cary, NC) was used for data analysis in this study. Chi-squared tests (χ^2^) tests were used to investigate the differences between cases and controls in terms of monthly income and selected medical co-morbidities including hypertension, diabetes, hyperlipidemia, obesity, a history of vasectomy, chronic prostatitis, alcohol abuse/alcohol dependence syndrome, and sexually transmitted diseases (STDs) within five years prior to index date. These medical co-morbidities are all potential risk factors for PC. We used conditional logistic regression analyses (conditioned on age group, urbanization level, geographic region, and index year) to calculate the odds ratio (OR) and corresponding 95% confidence interval (CI) as an estimation of association between PC and having been previously diagnosed with UC. However, after adding all the above mentioned medical co-morbidities into the regression model for adjustment, the alteration of the results was less than 10%, of the primary analysis model. Therefore, these co-morbidities were not included in the regression models. We used *p*≤0.05 for statistical significance in this study.

## Results

Of the total 2,900 cases and 14,500 controls, the mean age (± SD) was 70.2 years (± 11.05). Table 1 shows the results of the Pearson ^χ 2^ tests performed examining the differences in the distributions of monthly income and medical co-morbidities between cases and controls. After matching for age group, geographic region, and urbanization level, and index year, it shows that there were no significant differences in monthly income (*p* = 0.781) between cases and controls. In addition, there were no significant differences in the prevalence of the following co-morbidities: Hypertension, diabetes, hyperlipidemia, obesity, and alcohol abuse/alcohol dependence syndrome than controls. However, cases had a higher prevalence of STDs (*p* = 0.002), and chronic prostatitis (*p*<0.001) than controls.

**Table 1:** Demographic characteristics of subjects with prostate cancer and controls in Taiwan (n = 17,400).

Variable	Subjects with prostate cancer(*n = 2,900*)		Controls(*n = 14,500*)		P value
	Total No.	%	Total No.	%	
Monthly Income					0.781
No income	467	16.1	2,306	15.9	
NT$1–15,840	646	22.3	3,329	23.0	
NT$15,841–25,000	928	32.0	4,680	32.3	
≥NT$25,001	859	29.6	4,186	28.8	
Hyperlipidemia	1,101	38.0	5,270	36.3	0.085
Diabetes	937	32.3	4,586	31.6	0.427
Hypertension	1,913	66.0	9,629	66.4	0.211
Chronic prostatitis	241	8.3	513	3.6	<0.001
Obesity	14	0.5	59	0.4	0.564
Sexually transmitted disease	115	4.0	423	2.9	0.002
A history of vasectomy	55	1.9	218	1.5	0.120
Alcohol abuse	32	1.1	132	0.9	0.326

Table 2 presents the prevalence of prior UC between cases and controls. We found that 2,645 out of the 17,400 sampled subjects (15.2%) had received a diagnosis of UC prior to the index date; prior UC was found among 608 (21.0%) cases and 2,037 (14.1%) controls (*p*<0.001). Furthermore, conditional logistic regression analysis revealed that compared to controls, the OR of prior UC for cases was 1.63 (95% CI = 1.47–1.81)

**Table 2:** Odds ratios for previous urinary calculus according to stone location among the sample subjects (n = 17,400).

Variable	Total sample		Controls		Subjects with prostate cancer	
	No.	%	No.	%	No.	%
Presence of urinary calculus	2,645	15.2	2,037	14.1	608	21.0
OR (95% CI)	–		1.00		1.63*** (1.47–1.80)	
Presence of kidney calculus	664	4.3	506	3.6	158	6.5
OR (95% CI)	–		1.00		1.71*** (1.42–2.05)	
Presence of ureter calculus	305	2.0	254	2.0	51	2.2
OR (95% CI)	–		1.00		1.11 (0.82–1.51)	
Presence of bladder calculus	96	0.7	69	0.6	27	1.2
OR (95% CI)	–		1.00		2.06** (1.32–3.23)	
Presence of ≥ 2 locations of urinary calculus	943	6.0	719	5.5	224	1.2
OR (95% CI)	–		1.00		1.73*** (1.47–2.02)	
Presence of calculus, unspecified	637	4.1	148	6.1	489	3.8
Crude^ a^ OR (95% CI)	–		1.00		1.66*** (1.37–2.00)	

*Notes:* *** indicates *p*<0.001; ** indicates *p*<0.01; OR = odds ratio; Odds ratio was calculated by using conditional logistic regression (conditioned on sex, age group, urbanization level, and index year).

Table 2 further presents the ORs for prior UC among the sampled subjects by stone location. We categorized stone location into kidney, ureter calculus, bladder, unspecified, and ≥2 locations of UC. We found that cases were more likely to have been previously diagnosed with kidney calculus (OR = 1.71; 95% CI = 1.42–2.05), bladder calculus (OR = 2.06; 95% CI = 1.32–3.23), unspecified calculus (OR = 1.66; 95% CI = 1.37–2.00), and ≥2 locations of UC (OR = 1.73; 95% CI = 1.47–2.02) than controls. However, there was no significant relationship between PC and prior ureter calculus (adjusted OR = 1.11, 95% CI = 0.82–1.51).

Table 3 analyzed the relationship between PC and UC treatment method (extracorporeal shockwave lithotripsy (ESWL), endoscopic intervention, and percutaneous nephrolithotomy) by limiting the sampled subjects to those who all had previous diagnoses of UC. If a patient who had recurrent UC episodes may be treated differently, we only selected the most current therapeutic strategy for analysis. We found that of the patients with UC, none of the treatment methods for UC were significantly associated with risk of PC.

**Table 3:** Odds ratios for previous treatment procedures among the sampled subjects with urinary calculus.

Variable	Total sampleN = 2,645		ControlsN = 2,037		Subjects with prostate cancer N = 608	
	No.	%	No.	%	No.	%
Patients treated with extracorporeal shockwave lithotripsy	496	18.8	393	19.3	103	16.9
OR (95% CI)	–		1.00		0.80 (0.63–1.02)	
Patients receiving endoscopic intervention	306	11.6	240	11.8	66	10.9
OR (95% CI)	–		1.00		0.88 (0.66–1.18)	
Patients receiving percutaneous nephrolithotomy	73	2.8	55	2.7	18	3.0
OR (95% CI)	–		1.00		1.14 (0.66–1.95)	

*Notes:* OR = odds ratio; Odds ratio was calculated by using conditional logistic regression (conditioned on sex, age group, urbanization level, and index year).

Table 4 further explores the relationship between PC and prior diagnosed UC by the presence of a history of STD or chronic prostatitis. Multinomial logistic regression showed that prior diagnosed UC was associated with PC with (OR = 1.63, 95% CI = 1.03–2.57) or without (OR = 1.66, 95% CI = 1.49–1.84) the presence of STD, respectively, when compared to controls. In addition, prior diagnosed UC was associated with PC regardless of the presence of chronic prostatitis

**Table 4:** Odds ratios for previously diagnosed urinary calculus among the sample subjects by the presence of chronic prostatitis or sexually transmitted disease.

Exposure	Outcome ^a^	OR ^b^ (95% CI ^c^)^a^
Urinary calculus		
	Prostate cancer with sexually transmitted disease (*n* = 115)	1.63* (1.03–2.57)
	Prostate cancer without sexually transmitted disease (*n* = 2,785)	1.66*** (1.49–1.84)
	Controls (*n* = 14,500)	1.00
Urinary calculus		
	Prostate cancer with chronic prostatitis disease (*n* = 241)	2.00*** (1.66–2.47)
	Prostate cancer without chronic prostatitis disease (*n* = 2,659)	1.56*** (1.39–1.76)
	Controls (*n* = 14,500)	1.00

*Notes:* ^a^ Multinomial logistic regression model, comparing prostate cancer with sexually transmitted disease and prostate cancer with sexually transmitted disease to controls. ^b^ OR  =  odds ratio. ^c^ CI  =  confidence interval ; *** indicates *p*<0.001; * indicates *p*<0.05

## Discussion

This study succeeded in identifying an association between PC and a prior diagnosis of UC. Our finding supported and built upon the earlier study by Pelucchi et al [15], which found that the risk for PC was increased for those reporting urinary tract stones. Furthermore, after analyzing by stone location, we found that PC was associated with kidney calculus (OR = 1.71), bladder calculus (OR = 2.06), unspecified calculus (OR = 1.66), and ≥2 locations of UC (OR = 1.73). However, the association between PC and prior ureter calculus was not statistically significant.

UC has two etiological characterizations, the primary being metabolic and the secondary including anatomic, infectious, medication related, and disease related causes. Previous studies have established the association between UC and metabolic syndrome (MS), which is characterized by a cluster of features including dyslipidemia, hyperglycemia, hypertension, obesity, and insulin resistance [16]. Such symptoms have been demonstrated in a rat model of metabolic syndrome to precipitate changes in urinary constituents and to lead to an increased risk of uric acid and calcium stone formation [17]. One prospective longitudinal study in humans has demonstrated that uric acid stone formers have a significantly higher prevalence of diabetes and glucose intolerance, and an elevated serum triglyceride concentration compared to normal controls [18]. As the metabolic derangements accompany MS have been demonstrated to cause chronic low-grade inflammation and to contribute to the pathogenesis of PC [19], [20], it is possible that this is one underlying factors that can be used to explain the associations detected in this study.

Prior epidemiological studies have also provided strong evidence that environmental and dietary factors are important in accelerating the transition between latent and clinically apparent PC. For example, Japanese men experience an increase in the incidence of clinically apparent PC when they move to Hawaii [21], and African men have a much lower incidence of PC than do African-Americans [22]. Other studies have demonstrated that a rise in the rate of clinically apparent PC is associated with increased fat intake. They further suggested that this increase may be facilitated by elevated level of androgen production associated with increased fat intake [20], [22], [23]. Thus, it is also possible that dietary factors that contributed to urinary changes promoting stone formation also drove hormonal changes which accelerated the pathogenesis of PC.

This study’s strengths include the use of a population-based dataset, which enabled us to trace of all the cases of UC and PC during the study period. The large sample size afforded a considerable statistical advantage in detecting real differences between the two cohorts.

Nevertheless, this study suffered from several limitations that should be addressed. The first limitation is that the diagnoses of both UC and PC relied on administrative claims data reported by physicians and hospitals. These data may be less accurate than diagnoses made according to standardized criteria. Furthermore, as there is no linkage available between this database and the cancer registry, we did not have access to information regarding the stage or grade of PC. This precluded us from conducting any analysis taking these measures into consideration.

Second, some patient information on factors which may have had an effect on the associations detected in this study were not available through the administrative dataset. Some of these factors include tobacco use, alcohol and betel quid consumption, dietary habits, and body mass index.

Third, like many epidemiological studies, this investigation may have been victim to a surveillance bias in which patients with one condition (UC) were more likely to be diagnosed with a separate and possibly unrelated condition (PC) purely based on their increased exposure to the medical community. Therefore, it is possible that more PC cancer was detected among patients with UC due to increased imaging, which may have contributed to the association detected in this study. However, in this study the association between PC and a prior diagnosis of ureter calculus was not significant, discounting the possibility that surveillance bias heavily impacted our results.

Fourth, as this was a case-control study we were unable to comment on causality and were only able to report an association between PC and prior UC.

This investigation detected an association between PC and prior UC after adjusting for co-morbid medical disorders and socioeconomic factors. These results add to the evidence regarding the association between PC and prior UC and highlight a potential target population for PC screening. In addition, it is the hope of the authors that future prospective studies be conducted to establish temporality and to report risk estimates, as well as to elucidate any possible underlying mechanisms between these two conditions
